# Recognition and processing of double-stranded DNA by ExoX, a distributive 3′–5′ exonuclease

**DOI:** 10.1093/nar/gkt495

**Published:** 2013-06-14

**Authors:** Tianyu Wang, Han-Li Sun, Fang Cheng, Xian-En Zhang, Lijun Bi, Tao Jiang

**Affiliations:** ^1^National Key Laboratory of Biomacromolecules, Institute of Biophysics, Chinese Academy of Sciences, 15 Datun Road, Chaoyang District, Beijing 100101, China, ^2^Graduate School of Chinese Academy of Sciences, Beijing 100039, China and ^3^State Key Laboratory of Virology, Wuhan Institute of Virology, Chinese Academy of Sciences, Wuhan 430071, China

## Abstract

Members of the DnaQ superfamily are major 3′–5′ exonucleases that degrade either only single-stranded DNA (ssDNA) or both ssDNA and double-stranded DNA (dsDNA). However, the mechanism by which dsDNA is recognized and digested remains unclear. Exonuclease X (ExoX) is a distributive DnaQ exonuclease that cleaves both ssDNA and dsDNA substrates. Here, we report the crystal structures of *Escherichia coli* ExoX in complex with three different dsDNA substrates: 3′ overhanging dsDNA, blunt-ended dsDNA and 3′ recessed mismatch-containing dsDNA. In these structures, ExoX binds to dsDNA via both a conserved substrate strand-interacting site and a previously uncharacterized complementary strand-interacting motif. When ExoX complexes with blunt-ended dsDNA or 5′ overhanging dsDNA, a ‘wedge’ composed of Leu12 and Gln13 penetrates between the first two base pairs to break the 3′ terminal base pair and facilitates precise feeding of the 3′ terminus of the substrate strand into the ExoX cleavage active site. Site-directed mutagenesis showed that the complementary strand-binding site and the wedge of ExoX are dsDNA specific. Together with the results of structural comparisons, our data support a mechanism by which normal and mismatched dsDNA are recognized and digested by *E. coli* ExoX. The crystal structures also provide insight into the structural framework of the different substrate specificities of the DnaQ family members.

## INTRODUCTION

DNA replication, repair and recombination often require 3′–5′ exonucleases to excise nucleotides from the 3′ termini of DNA. It has been reported that 3′–5′ exonucleases play an essential role in DNA repair pathways by removing damaged nucleotides. These exonucleases also remove normal nucleotides during processes such as genetic recombination to generate the appropriate 3′ termini for the subsequent steps of DNA metabolism (1–3).

The DnaQ family is one of the major 3′–5′ exonuclease superfamilies and is conserved from *Escherichia coli* to *Homo sapiens*. Its members, such as the DNA polymerase I Klenow fragment (KF), the ε subunit of DNA polymerase III, Werner syndrome protein, exonuclease I (ExoI), exonuclease X (ExoX), three prime repair exonuclease 1/2 (TREX1/2), etc., participate in various types of nucleotide acid metabolism, including DNA replication, DNA repair, homologous recombination, DNA transcription and some RNA processing systems (4,5). DnaQ family members share a conserved cleavage active site, which contains three important ‘Exo’ motifs, consisting of four conserved acidic residues (three aspartate residues and one glutamate residue) as well as either a histidine(h) or tyrosine(y) residue, which divides the DnaQ superfamily into the following two families: DEDDh and DEDDy. The histidine or tyrosine residues work with two divalent metal ions, such as Mg^2+^ or Mn^2+^, to form a complete 3′ terminal cleavage active site (6–9).

Despite sharing a common catalytic mechanism of hydrolysis, members of DnaQ family have been reported to display distinct substrate specificities. For example, ExoI, a processive 3′–5′ exonuclease implicated in DNA recombination and repair pathways, can only digest single-stranded DNA (ssDNA) and shows no significant hydrolysis activity against double-stranded DNA (dsDNA) (10,11). Similarly, the ε subunit of polymerase III acting alone can only digest ssDNA, but in combination with other polymerase III subunits, it performs proofreading of dsDNA (11). KF, a large fragment of polymerase I, contains a 3′–5′ exonuclease domain with a DnaQ fold (Exo domain) and a 5′–3′ polymerase domain. Although KF performs proofreading of mismatched dsDNA, its crystal structure in complex with mismatched dsDNA indicates that one strand of the mismatched dsDNA binds to the Exo domain, and the other strand binds to the KF polymerase domain (12), suggesting that the polymerase domain is indispensible for dsDNA recognition.

In contrast to the three above-mentioned exonucleases, some other DnaQ members, including TREX1/2 (the major nuclear DNA-specific 3′–5′ exonuclease in mammalian cells) and ExoX, are capable of using both ssDNA and dsDNA as substrates (13–15), despite only containing a single exonuclease domain.

The crystal structure of TREX1 in complex with ssDNA indicates a single-stranded digestion mechanism, similar to other DnaQ members (16). However, due to the lack of a complex crystal structure of TREX1 bound to dsDNA, the double-stranded recognition and processing mechanisms of TREX1/2 and other members of the DnaQ family remain elusive.

ExoX is one of several redundant exonucleases involved in the mismatch repair system, UV repair, homologous recombination and the stabilization of tandem repeats in *E. coli* (1,2,17–19). Like TREX1/2, ExoX contains only an exonuclease domain; however, it can act on both ssDNA and dsDNA in a distributive manner and digests only one or two nucleotides per reaction cycle (15). To elucidate the mechanism by which dsDNA is recognized and processed by single-domain DnaQ members, such as ExoX and TREX1/2, we determined the structures of ExoX in complex with three distinct dsDNA substrates: 3′ overhanging duplex DNA, a 12 bp blunt-ended dsDNA and 5′ overhanging partially mismatched duplex DNA, referred to here as complexes I, II and III, respectively. The crystal structures obtained revealed that ExoX interacts with dsDNA via two DNA-binding sites, one for the substrate strand and another for the complementary strand. In the blunt-ended DNA complex II and 5′ overhanging DNA complex III structures, a ‘wedge’ composed of the ExoX Leu12 and Gln13 residues penetrates between the first two base pairs of the 3′ end of the dsDNA to break the terminal base pairing, resulting in precise feeding of the substrate 3′ terminus into the cleavage active site. Analysis of site-directed mutations via activity assays demonstrated that the complementary strand binding and the wedge of ExoX are dsDNA specific. Moreover, structural comparisons indicated that the complementary strand-binding motif only exists in DnaQ members that possess dsDNA digestion activity, such as TREX1. These results suggest a working model for the digestion of both normal and mismatched dsDNA by ExoX and establish a structural framework for the different substrate specificities of DnaQ family members.

## MATERIALS AND METHODS

### Protein expression and purification

The full-length ExoX (220 aa) gene was amplified via polymerase chain reaction from *E. coli* strain K12 genomic DNA and cloned into the pQE30 plasmid (Qiagen, Germany). A truncated ExoX gene (residues 1–167) was cloned into pET24a (Novagen, Germany). Full-length and truncated ExoX were both expressed in BL21 *E. coli* cells. The cells were cultured to an OD of 0.6 in LB medium at 37°C, cooled to 16°C and induced with 0.2 mM isopropyl 1-thio-β-d-galactopyranoside for 13 h, then harvested, lysed in buffer A (20 mM Tris-HCl [pH 8.0], 500 mM NaCl, 20 mM imidazole) and centrifuged. The resulting cleared supernatants were loaded onto Ni-NTA resin (GE Healthcare, USA) and purified using a standard protocol. The eluted proteins were further purified using Superdex 200 gel filtration columns (GE Healthcare, USA) with buffer B (20 mM Tris–HCl, 100 mM NaCl, 5 mM EDTA). The obtained protein was concentrated to 8 mg/ml for crystallization. SeMet-substituted ExoX was overexpressed as previously described and purified as indicated above (20). For biochemical analyses, full-length ExoX was dialyzed in buffer C (25 mM Tris [pH 7.6], 250 mM NaCl, 50% glycerol).

### Crystallization and radiographic structure determination

Oligonucleotides were purchased from Augct Bio Ltd. (Beijing, China). For the 3′ overhanging dsDNA substrate, two 17-mer oligonucleotides (5′-CGGATCCACAATGACCT-3′ and 5′-GTCATTGTGGATCCGAG-3′) were annealed to create a 17 bp dsDNA with a 3′ dinucleotide overhang. For the blunt-ended dsDNA substrate, two 12-mer oligonucleotides (5′-CTCGAATCTACA-3′ and 5′-TGTAGATTCGAG-3′) were annealed to generate a 12 bp blunt-ended dsDNA. For the 3′ recessed mismatch-containing dsDNA substrate, the same 17-mer oligonucleotide from one strand of the 3′overhanging dsDNA (5′-GTCATTGTGGATCCGAG-3′) was self-annealed to form a DNA duplex with a 5 nt 5′ overhang and mispaired 3′ terminal nucleotides.

For crystallization, ExoX was incubated with dsDNA at a ratio of 1:0.6 for 30 min at 4°C, and the resultant protein–DNA complexes were purified using Superdex 200 gel filtration columns. For crystallization of the Se-Met-derivative ExoX in complex with 3′ overhanging dsDNA (complex I), purified complexes were concentrated to 2 mg/ml. ExoX in complex with blunt-ended dsDNA (complex II) and ExoX in complex with 3′ recessed mismatch-containing dsDNA (complex III) were concentrated to 1 mg/ml. All crystals were grown via the hanging-drop vapor diffusion method at 16°C using 0.1 M Tris–HCl (pH 8.0), 0.2 M NaCl and 20% PEG 3350 for complex I and 0.1 M MES (pH 6.3) and 17% PEG 6000 for complexes II and III. Individual rod-shaped crystals of all three complexes were obtained through microseeding.

Before data collection, crystals were transferred to a reservoir solution containing 25% PEG 3350 and 20% sucrose and flash frozen in liquid nitrogen. All radiographic data sets were collected on beamline BL17U at the Shanghai Synchrotron Radiation Facility (SSRF, Shanghai, China) and processed using HKL2000 (21). For the Se-Met derivative complex I, the phase was determined via single-wavelength anomalous dispersion. An electron density map and initial model were built using PHENIX (22). Further model building was performed with COOT (23), and refinement was conducted using REFMAC (24) and PHENIX-Refine (22). For complexes II and III, the structures were solved via molecular replacement and refined using REFMAC and PHENIX-Refine. Data collection and refinement statistics are presented in Supplementary Table S1.

### Mutagenesis and exonuclease assay

Site-directed mutations were created in full-length ExoX using the Quikchange procedure (Stratagene, USA) and confirmed by DNA sequencing. Mutated proteins were expressed and purified using the protocols described above. Standard exonuclease reactions contained 10 mM Tris–HCl (pH 8.0), 10 mM MgCl_2_, 1 mM NaCl, 2 mg/ml bovine serum albumin, 32 nM of an 1893 bp dsDNA and an appropriate amount of full-length ExoX. The reaction mixtures were incubated at 37°C for 10 min, quenched by the addition of 10 mM EDTA, 0.1% sodium dodecyl sulphate and 150 μg/ml proteinase K and incubated at 42°C for 30 min. To visualize the dsDNA digestion results, the reaction products were resolved on 1% agarose gels and stained using ethidium bromide. To determine whether the second DNA-binding site and the wedge were dsDNA-specific, we used the 17 nt ssDNA (5′-CGGATCCACAATGACCT-3′) and the 17 bp dsDNA (5′-CGGATCCACAATGACCT-3′ and 5′-AGGTCATTGTGGATCCG-3′) as substrates to measure the excision activity of wild type and site-directed mutants of ExoX using the reaction solution described above. Reactions were initiated and quenched as described above. The remaining substrate was quantified with the Quant-iT™ OliGreen ssDNA Reagent and Quant-iT™ PicoGreen dsDNA Reagent (Invitrogen, USA). The resultant fluorescence was quantified using a Varioskan Flash Multimode Reader (Thermo, USA).

## RESULTS

### Structure of ExoX in complex with 3′ overhanging dsDNA (complex I)

**Figure 1.**
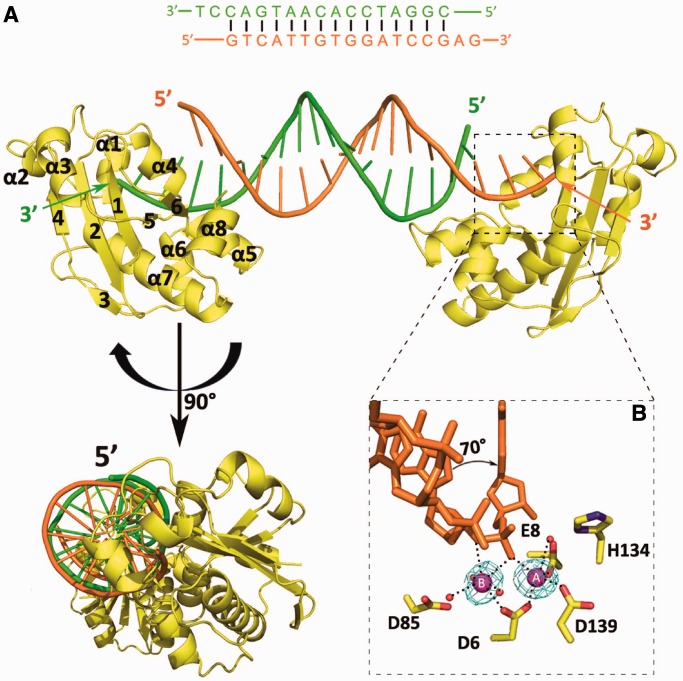
Overall structure of complex I. (**A**) Ribbon diagram of ExoX in complex with 3′ overhanging dsDNA (ratio of 2:1). (**B**) Close-up view of the active site of ExoX; the DNA scissile strand twists and inserts into the active cavity of ExoX. Strong density peaks are observed at the two conserved metal-binding sites of the DnaQ family, suggesting the location of the two metal ions in ExoX. The difference (Fo-Fc) maps are contoured at 2.5σ.

Although attempts to crystallize full-length ExoX failed, we were able to crystallize and solve the crystal structure of a C-terminal truncation of ExoX containing residues 1–167 (hereafter referred to as ExoX, Supplementary Figure S1), which retained activity. In each asymmetrical unit, two protein molecules (A and B) bind to the ends of a single dsDNA molecule, thus forming a dumbbell-like structure (Figure 1A). Each monomer of ExoX forms a bean-shaped core consisting of a central β-sheet (strands β1–β5) surrounded by eight α-helices (α1–α8), with helices α3–α4 on one side and helices α1–α2 and α5–α8 on the other side (Figure 1A). Analysis of the ExoX structure using the Dali server showed that ExoX has the highest degree of similarity to the ε subunit of DNA polymerase III (Z-score of 18.6, PDB code: 1J53). Despite these proteins sharing only ∼33% sequence identity, ExoX can be superimposed on the ε subunit of DNA polymerase III with a root-mean-square deviation for the Cα atoms of only 1.9 Å. This similarity, together with the presence of all three Exo motifs in ExoX (Supplementary Figure S2), provided further confirmation that ExoX belongs to the DnaQ family. The active site of ExoX consists of four conserved acidic residues (Asp6 and Glu8 in β1, Asp85 in α4, Asp139 in α7) and His134 from the loop between α6 and α7, all of which participate in the cleavage process (Figure 1B).

**Figure 2.**
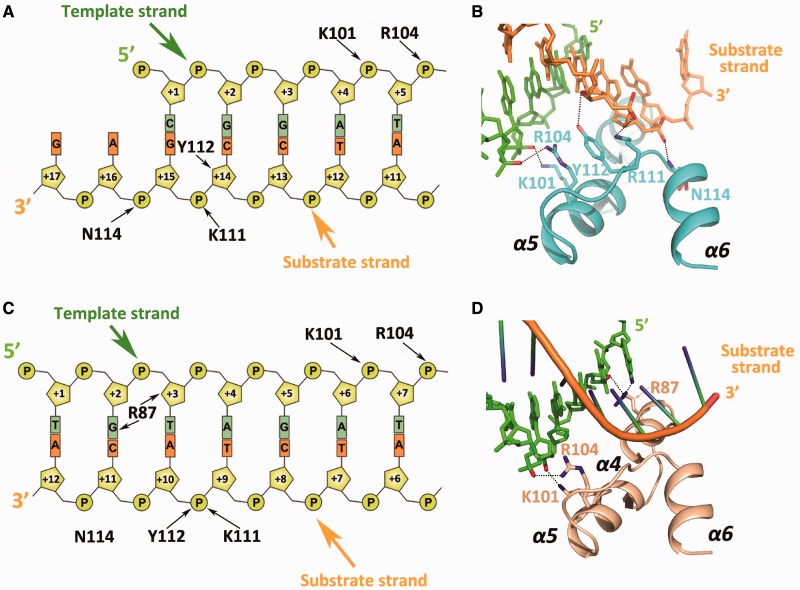
Protein–DNA interactions in complexes I and II. Schematic diagram of the protein–DNA interactions in complexes I (**A**) and II (**C**). (**B**) Close-up view of the five interactions between ExoX and the 3′ overhanging dsDNA. (**D**) Close-up view of the three interactions between ExoX and the blunt duplex DNA complementary strand. Interactions exist between Arg87 and the complementary DNA strand in complex II that cannot be observed in complex I.

In complex I, the 3′ overhang is bent at an angle of 70 degrees between the first two nucleotides and extends into the active site (Figure 1B). Outside the active site, a substrate DNA strand-binding site, mainly composed of Lys111, Tyr112 and Asn114 in the α5–α6 loop, interacts with the sugar–phosphate backbone of the substrate strand (Figures 2A and B). This site is similar to the ssDNA binding sites observed in other DnaQ family members (16). Moreover, Lys101 and Arg104 of α5 form a specific positively charged binding site for the complementary strand. Most of the residues formed electrostatic interactions or hydrogen bonds with the DNA sugar–phosphate backbone, while few displayed electrostatic interactions or hydrogen bonding with DNA bases, which is indicative of a non–sequence-specific DNA-binding site (Figures 2A and B). Strong density peaks were observed at the two conserved metal-binding sites of the DnaQ family, A and B (8,25) (Figure 1B). The intact substrate observed in our structure suggest that the metals involved are unlikely to be active metals (such as Mn^2+^ or Mg^2+^), and are probably inactive metals (such as Ni^2+^ or Na^+^). Although we cannot be certain of the exact metal ion species present, the undefined metal ion is of no consequence for the following discussion.

The ion bound at site A (MeA) is penta-coordinated by the side-chain oxygen atoms of Asp6, Glu8 and Asp139, one solvent water molecule and an oxygen atom of the substrate phosphate group, while the ion bound at site B (MeB) is hexa-coordinated by the side-chain oxygen atom of Asp6, three solvent water molecules and two oxygen atoms of the substrate phosphate group. The coordination of the two metal ions in ExoX is similar to that of the other members of the DEDDh subfamily, suggesting a classical phosphodiester bond cleavage mechanism for ExoX (8,26).

### Structure of the ExoX in complex with blunt-ended DNA (complex II)

Our structure of the blunt-ended DNA complex is the first to show the specific interaction of a DnaQ member with a blunt-ended dsDNA. Similar to complex I, two ExoX monomers bind to the individual ends of one DNA molecule. However, several significant differences were observed between these two complex structures. First, in addition to the two residues that interacted with the complementary strand and were observed in the 3′ overhanging DNA complex (Lys101 and Arg104), an adjacent positively charged Arg87 residue was also involved in complementary strand binding via an interaction with the +2 and +3 nucleotides of the 5′ terminus (Figures 2C and D). This suggests that ExoX forms more protein–DNA interactions with dsDNA containing a single-nucleotide 3′ overhang or blunt-ended dsDNA than with dsDNA containing a two-nucleotide or longer 3′ overhang.

Second, the coordination of MeA in complexes I and II is different. Compared with the penta-coordination observed in complex I, MeA is tetra-coordinated in complex II, with one proposed water nucleophile missing, accompanied by a slight reorientation of the His134 side chain closer to the substrate phosphate. This difference may be attributed to the pH at which the crystals were grown (8.0 for complex I and 6.3 for complex II) (Supplementary Figure S3).

**Figure 3.**
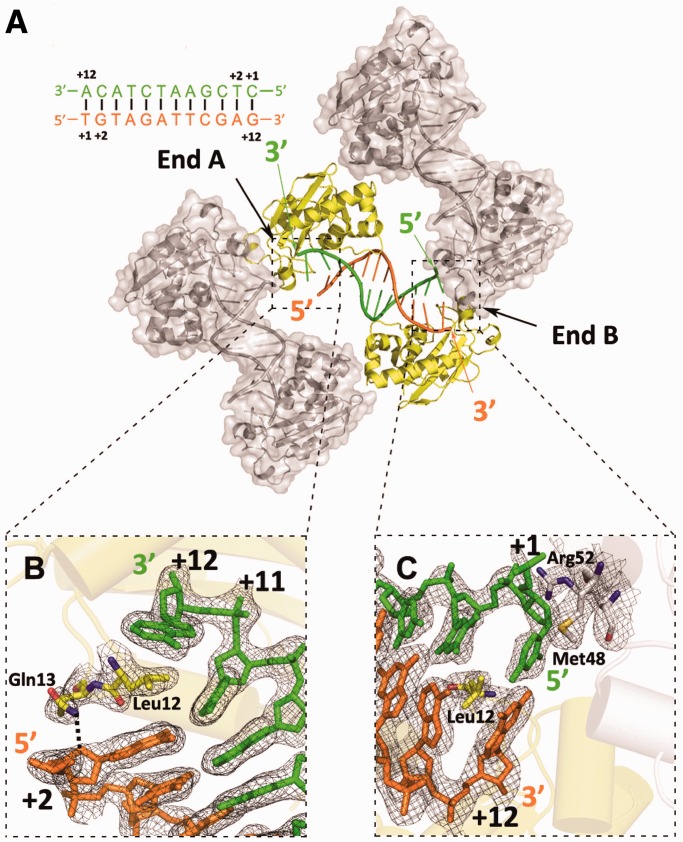
Structural differences in the two dsDNA ends in complex II. (**A**) Lattice packing creates two different dsDNA ends, termed A and B. (**B**) Close-up view of dsDNA end A, showing that the Leu12 and Gln13 wedge breaks the last base pair and distorts the penultimate base pair. (**C**) Close-up view of dsDNA end B, showing that Met48 and Arg52 of an adjacent symmetrical ExoX molecule (gray) interact with the terminal 5′ nucleotide.

Third, at both DNA ends (A and B) in complex II, a wedge composed of two key residues (Leu12 and Gln13) located in the loop between β1 and β2 (loop 1) was observed. Leu12 penetrates between the two terminal base pairs, while Gln13 is inserted halfway between the two terminal base pairs and forms an interaction with the second nucleotide of the 5′ terminus. The wedging of Leu12 and Gln13 results in a distortion of the first base pair and facilitates full insertion of the first substrate nucleotide into the active center. Interestingly, ends A and B were found to adopt different conformations (Figure 3). While the 5′ terminal nucleotide at end A was invisible in the electron density map, the 5′ terminal nucleotide at end B was clearly observed to pair with the 3′ terminal nucleotide, although this base pair was largely distorted. The conformational difference between the two DNA ends can likely be attributed to their different lattice-packing environments. At end B, the 5′ terminus is surrounded by α1 and the loop between α1 and α2 (loop 2) of an adjacent symmetric molecule and is stabilized by interactions with Met48 of α1 and Arg52 of loop 2, possibly restricting its movement (Figure 3C). In contrast, end A does not display stacking with the symmetric molecule, and thus, the 5′ terminal nucleotide is unrestricted (Figure 3B), likely reflecting a free conformation in solution. Therefore, the structure of complex II suggests that ExoX unwinds the first base pair of the blunt-ended dsDNA and feeds the 3′ end of the substrate strand into the active center.

### Structure of ExoX in complex with 5′ overhanging DNA (complex III)

**Figure 4.**
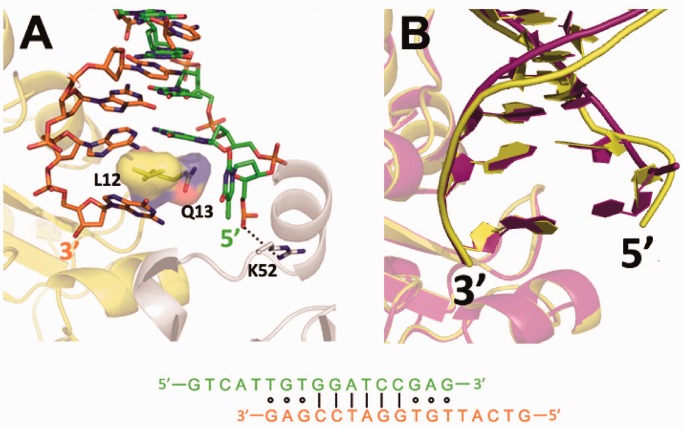
Structural overlay of dsDNA end B in complexes II and III. (**A**) Arg52 from the adjacent symmetrical molecule interacts with the oxygen atom of the 5′ terminal phosphate group at end B in complex III. (**B**) Structural overlay showing that in complex III, the end B base pair is broken, differing completely from what is observed in complex II, which displays a twist but also an intact base pair. Complex III is shown in yellow and complex II in magenta.

To investigate the differences between the digestion mechanisms for mismatched and paired DNA, we attempted to solve the structure of ExoX in complex with mismatched DNA. To mimic the excision reaction in the mismatch repair system and diminish the effects of intermolecular packing due to an adjacent crystallographically symmetrical molecule observed near end B of blunt-ended complex II, we designed a partially paired 17 nt self-complementary dsDNA containing a 5 nt overhang at the 5′ terminus. In the remaining 12 nt of each strand, the central 6 paired nucleotides are flanked by 3 mispaired nucleotides at both the 3′ and 5′ ends. The ExoX-5′ overhanging DNA complex (complex III) structure was solved by molecular replacement. The density of the 5 nt overhang region at the 5′ DNA terminus was missing in the structure, most likely due to flexibility resulting from the absence of protein interactions. Moreover, the first base pair is broken at end A, and the complementary nucleotide (+1 nt of the 5′ terminus) is also invisible, identical to complex II structure. Most of the protein–DNA interactions in complex III are the same as in complex II, with a few exceptions. Arg87 interacts simultaneously with the +2 and +3 nt of the 5′ terminus of the complementary strand in complex II, whereas Arg87 only interacts with the +2 nt of the 5′ terminus of the complementary strand in complex III, confirming the existence of a complementary strand binding site that includes Arg87, Lys101 and Arg104. Additionally, while only the first base pair was distorted in complex II, the first mismatched base pair of the 5′ overhanging DNA in complex III is completely broken, suggesting that mismatched pairs with a 5′ overhang can be broken more easily than intact base pairs (Figure 4B). This observation implies that ExoX may have proofreading or end-cleansing ability. Considering that some polymerases responsible for non-replicative synthesis lack 3′–5′ exonuclease activity, the putative proofreading role of ExoX may be exploited by cells to increase non-replicative synthesis efficiency.

### Activity analysis of ExoX mutations

**Figure 5.**
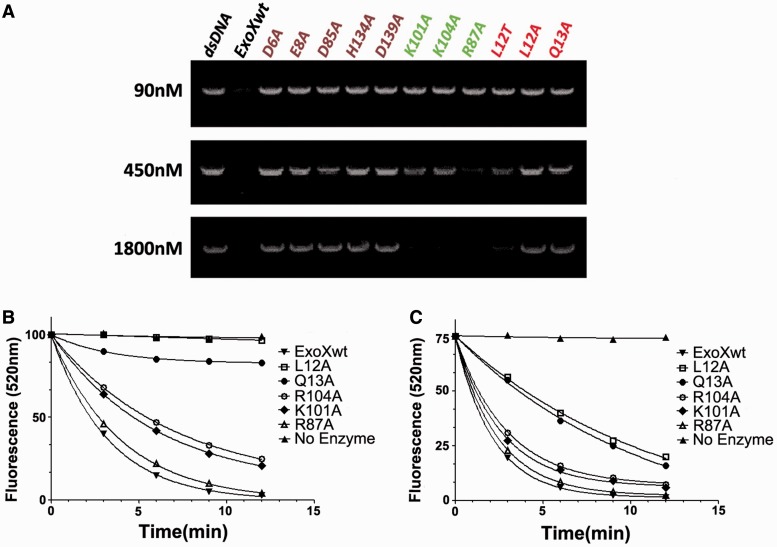
Mutant ExoX enzyme assays. (**A**) Cleavage of dsDNA by wild-type (WT) and mutant ExoX. The indicated concentrations of wild-type and mutated ExoX were incubated with a 32 nM concentration of an 1893 bp dsDNA substrate at 37°C for 10 min, and the products were then separated on agarose gels and stained with ethidium bromide. (**B**) Time course experiments for dsDNA cleavage by wild-type (WT) and mutant ExoX. The concentration of a 17-bp dsDNA substrate was 50 nM. The concentration of WT ExoX was 1 nM while that of ExoX mutants was 10 nM. (**C**) Time course experiments for ssDNA cleavage by WT and mutant ExoX. The concentration of a 17 mer ssDNA substrate was 100 nM. The concentration of WT ExoX was 1 nM, while that of ExoX mutants was 3 nM (see ‘Materials and Methods’ section for details). The amount of dsDNA (B) and ssDNA (C) remaining was determined by measuring light emission at 520 nm and fitting with a first-order decay model.

To verify our structural findings, we tested the exonuclease activities of mutants harboring point mutations in the 3′ complementary strand-interacting residues (Arg87, Lys101 and Arg104), the five active site residues (Asp6, Glu8, Asp85, His134 and Asp139) and the two base pair-unwinding residues (Leu12 and Gln13). Exonuclease activities of wild-type ExoX (ExoX^wt^) and the mutants toward dsDNA were tested using an 1893 bp dsDNA substrate and assessed via agarose gel electrophoresis and ethidium bromide staining (Figure 5A). The D6A, E8A, D85A, H134A and D139A mutants showed no detectable digestion activity, even at the highest concentrations tested, consistent with the role of these highly conserved residues in the DnaQ family two-metal digestive mechanism. The dsDNA digestive activity of K101A and R104A, which interact with the phosphate backbone of the complementary strand of the substrate DNA, were modestly decreased compared with ExoX^wt^. The enzyme activity of R87 was also clearly reduced, though to a lesser extent, supporting the role of Arg87 residue in dsDNA recognition and digestion. Furthermore, the L12A and Q13A mutations resulted in total inhibition of enzyme activity, verifying the role of these residues in dsDNA unwinding. Intriguingly, mutation of Leu12 to threonine also significantly diminished enzyme activity, suggesting that the hydrophobic nature of the Leu12 side chain, in addition to its size, is functionally important.

The results obtained suggest that Arg87, Lys101, Arg104, Leu12 and Gln13 are crucial for dsDNA digestion. To evaluate the dsDNA specificity of these residues, we measured the enzyme activity of ExoX^wt^ and these mutants using the 17 bp dsDNA and 17 nt ssDNA substrates. The K101A and R104A mutations resulted in a ∼5-fold decrease in ssDNA digestion activity and a 20-fold decrease in dsDNA digestion activity relative to ExoX^wt^. The digestion activity of the R87A mutant was reduced by ∼3-fold for ssDNA and ∼10-fold for dsDNA. These data are consistent with our observation that K101, R104 and R87 are involved in dsDNA binding and suggest that these amino acids play unique roles in the dsDNA digestion process. The L12A and Q13A mutants exhibited the most pronounced differences in digestion activity for ssDNA and dsDNA: their ssDNA digestion activity was reduced by ∼10-fold relative to ExoX^wt^, and both exhibited >60-fold decrease in dsDNA digestion activity. This major decrease in excision activity suggests that Leu12 and Gln13 play a key role in dsDNA substrate digestion (Figure 5, Table1).

**Table 1.** Exonuclease activity of ExoX^wt^ and its variants on dsDNA and ssDNA

ExoX	dsDNA activity (nm Nt/s/nm E)	ssDNA activity (nm Nt/s/nm E)
Wild type	2.2 ± 0.4	4.6 ± 0.7
R87A	(2.5 ± 0.6) × 10^−1^	1.4 ± 0.5
K101A	(1.3 ± 0.4) × 10^−1^	1.2 ± 0.4
R104A	(1.1 ± 0.3) × 10^−1^	(9.9 ± 1.0) × 10^−1^
L12A	(4.7 ± 0.6) × 10^−3^	(3.5 ± 0.5) × 10^−1^
Q13A	(3.3 ± 0.5) × 10^−2^	(4.3 ± 0.9) × 10^−1^

The dsDNA and ssDNA activity assays were performed with ExoX^wt^ and its variants. The concentration of substrate, enzyme and its variants were the same as those in Figure 5. All activity values were calculated by Origin7.0 using first-order decay model. All data are presented as means ± S.D. (n = 3). nm Nt/s/nm E: nmol of nucleotide released/s/nmol of enzyme.

## DISCUSSION

Here, we report the crystal structures of ExoX in complex with three different DNA substrates. These structures contain the conserved substrate strand-binding site that has been previously identified in other DnaQ family members and reveal a previously uncharacterized complementary strand-binding site and a ‘wedge’ composed of Leu12 and Gln13 that separates the 3′ end of the dsDNA. Mutagenesis analyses indicate that the complementary strand-binding site and the wedge are dsDNA specific. Moreover, compared with the matched 3′ end, it is likely that the mismatched 3′ can be separated more easily.

We propose a mechanism for the excision of dsDNA with both paired and unpaired termini by ExoX. First, ExoX captures the DNA end via the DNA phosphate backbone-binding regions on the surface of ExoX. This double-binding mechanism prevents the DNA from slipping out of the ExoX protein before the reaction begins and helps to hold the substrate strand in the correct position and orientation. In addition to DNA capture, loop 1 (specifically Leu12 and Gln13) inserts into the space between the stacked base pair and prevents these bases reannealing. Gln13 holds the 5′ terminus of the complementary strand by interacting with the second nucleotide of the 5′ terminus to feed the substrate strand into the active center. Next, the scissile phosphate is cleaved, followed by the release of a mononucleotide along with the DNA product from the enzyme. The extensive DNA–ExoX interactions observed in complex III, and the different B ends in complex II and III suggested that ExoX has a strong capacity to handle mismatched ends, implying that ExoX has a proofreading and end-cleansing functional role that may be essential for increasing the efficiency of non-replicative synthesis.

**Figure 6.**
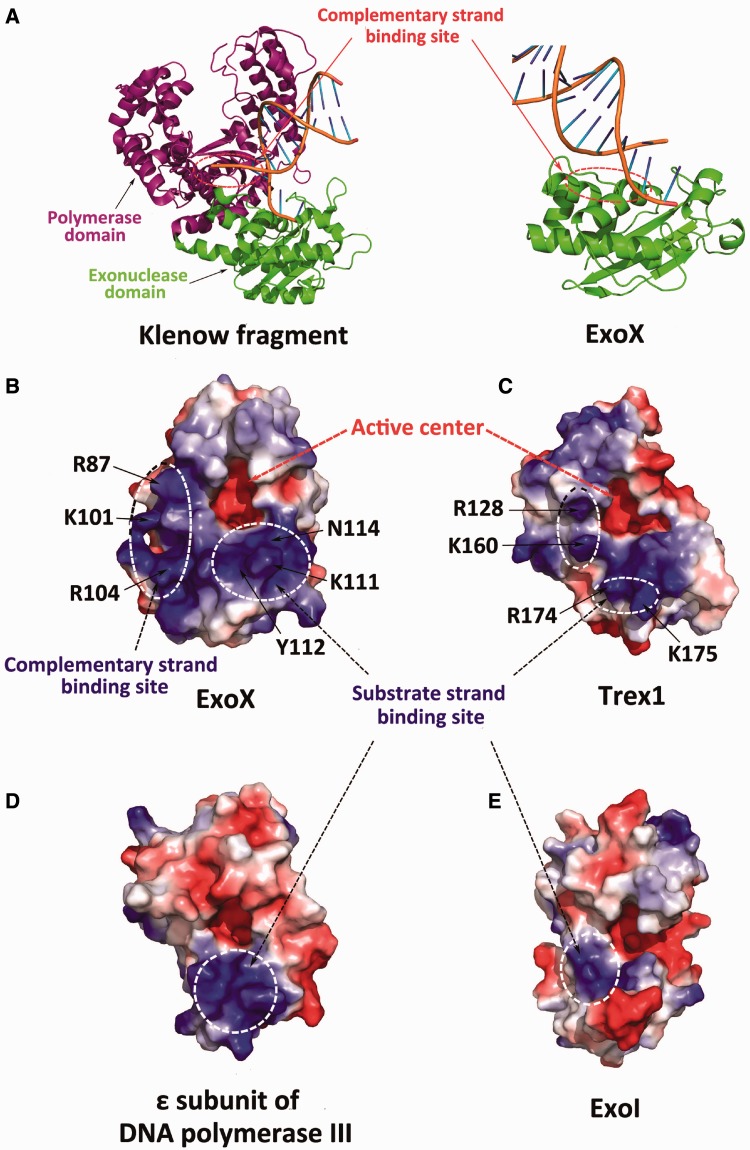
Structural comparison of the DnaQ family members. (**A**) The KF–dsDNA complex (left, PDB code: 1KLN) binds the complementary substrate DNA via its polymerase domain, not its exonuclease domain. In contrast, ExoX (right) interacts with the complementary substrate DNA via its exonuclease domain. **(B–E)** Surface electrostatic potential of ExoX (B), TREX1 (C, PDB code: 2O4I), the ε subunit of DNA polymerase III (D, PDB code:1J53) and ExoI (E, PDB code: 1FXX). Like ExoX, TREX1 (an exonuclease that digests dsDNA and ssDNA) contains a potential complementary strand-binding site, in addition to the substrate strand-binding site. However, ExoI and the ε subunit of DNA polymerase III, which only digest ssDNA, contain only the substrate strand-binding site.

Structural comparisons with other DnaQ family members further support these observations and reveal the structural basis for the substrate specificity of different DnaQ family members. Structural alignment indicated that the two DnaQ members that only display ssDNA activity, the Exo domain of KF and the ε subunit of DNA polymerase III, contain only the substrate strand-binding site (8,12). To perform the proofreading function, another domain (the 5′–3′ polymerase domain) is required for complementary DNA strand binding (Figure 6A), much as the alpha subunit is required for the ε subunit of DNA polymerase III to perform a similar function (11,12).

In contrast, TREX1, which also exhibits dsDNA digestion activity, contains a positively charged region equivalent to the DNA complementary strand-binding site of ExoX in addition to its substrate strand-binding site (16). Defective dsDNA digestion by TREX1 can lead to Aicardi-Goutières syndrome, systemic lupus erythematosus (neuropsychiatric SLE) (16) and familial chilblain lupus (27). Mutation of Arg128 in TREX1, which is equivalent to the Arg87 residue of ExoX, results in neuropsychiatric SLE. This outcome has been suggested to be due to the destabilization of dsDNA because mutation of Arg128 impairs the dsDNA digestion activity to a greater extent than that of ssDNA, and because Arg128, in a crystal structure of TREX1 complexed with a 4 nt ssDNA, is observed to interact with the substrate ssDNA (16,28,29). From our observations it is now clear that the Arg128 mutation likely reduces the ability of TREX1 to bind to the complementary strand of dsDNA (Figure 6).

**Figure 7.**
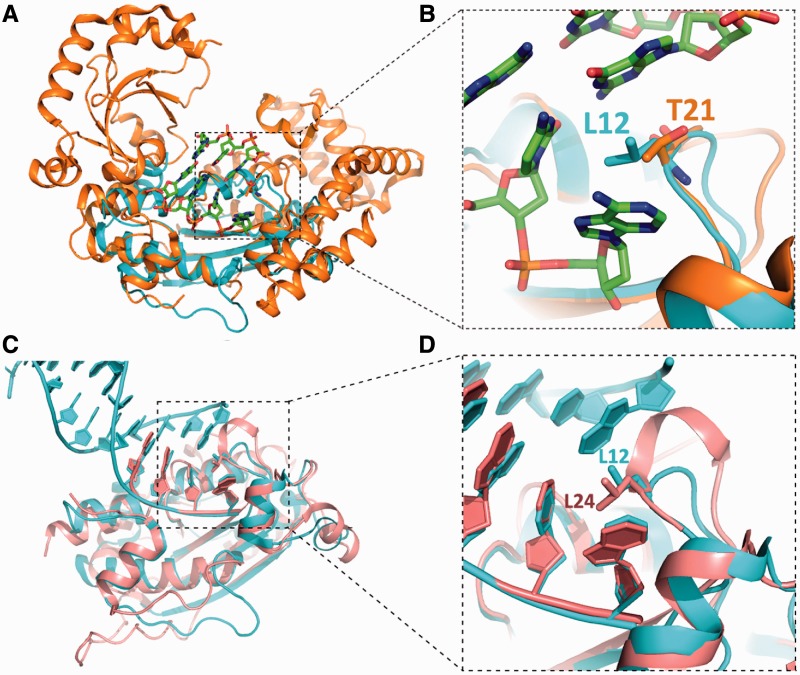
Structural comparison of ExoX with ExoI (PDB code: 1FXX) and TREX1 (PDB code: 2O4I). (**A**) Structural overlay of ExoX and ExoI. (**B**) The Leu12 and Gln13 amino acids of ExoX, which play crucial roles in base pair breaking, are replaced by Thr21 and His14 in ExoI. (**C**) Structural overlay of ExoX and TREX1. (**D**) Leu12 is highly conserved between TREX1 and ExoX.

ExoX digests ssDNA and dsDNA in a distributive manner, which can be attributed to the lack of the long ssDNA substrate-binding cavity observed in other processive DnaQ members, such as ExoI (30). Moreover, structural comparisons indicate that the wedge may prevent the 3′ terminus of the digestion product from moving forward into the active site without the release of the product from ExoX, especially when digesting blunt-ended or dsDNA with a 5′ overhang. This observation is in line with our structural mapping results, which showed that the Leu12 residue of ExoX is conserved in TREX1/2 but is replaced by a threonine in ExoI (Figure 7) (30).

The DnaQ family members are implicated in a variety of DNA recombination and repair pathways. However, the exact cellular function of many DnaQ family members remains elusive. Our results may aid in the design of future biochemical or structural analyses to further characterize the biological roles and regulatory mechanisms of these enzymes.

## ACCESSION NUMBERS

The atomic coordinates and structure factor amplitudes of all structures have been deposited in the Protein Data Bank with the accession numbers 4FZX, 4FZY and 4FZZ.

## SUPPLEMENTARY DATA

Supplementary Data are available at NAR Online: Supplementary Table 1, Supplementary Figures 1–3.

## FUNDING

National Natural Science Foundation of China [31021062, 31025009, 30970590]; National High Technology Research and Development Program of China [2011CB910302]; National Basic Research Program of China [2009CB825402]; Key Project Specialized for Infectious Diseases from the Chinese Ministry of Health [2008ZX10003-005]. Funding for open access charge: National Natural Science Foundation of China [31021062].

*Conflict of interest statement*. None declared.

## Supplementary Material

Supplementary Data
