# Supplementary material for: Recognition and processing of double-stranded DNA by ExoX, a distributive 3′–5′ exonuclease
Source: Nucleic Acids Res. 2013 Jun 14;41(15):7556–65. doi: 10.1093/nar/gkt495 (PMC3753628; doi:10.1093/nar/gkt495)
Supplement: Supplementary Data [file supp_41_15_7556__index.html]

Recognition and processing of double-stranded DNA by ExoX, a distributive 3′–5′ exonuclease — Recognition and processing of double-stranded DNA by ExoX, a distributive 3′–5′ exonuclease — Supplementary Data 

# Recognition and processing of double-stranded DNA by ExoX, a distributive 3′–5′ exonuclease

## Supplementary Data

files

**Files in this Data Supplement:**

- Supplementary Data - pdf file
