# Supplementary material for: Recognition and processing of double-stranded DNA by ExoX, a distributive 3′–5′ exonuclease
Source: Nucleic Acids Res. 2013 Jun 14;41(15):7556–65. doi: 10.1093/nar/gkt495 (PMC3753628; doi:10.1093/nar/gkt495)
Supplement: Supplementary Data [file supp_gkt495_nar-01014-2013-File009.pdf]

Recognition and processing of double-stranded DNA by ExoX , a distributive 3' – 5' exonuclease

Tianyu Wang, Han-Li Sun, Fang Cheng, Xian-En Zhang, Lijun Bi, and Tao Jiang

SUPPLEMENTARY DATA

Table S1	Statistics of data collection and refinement
Figure S1.	Enzyme assays demonstrated that NT (NT167, residues 1-167) had a comparable dsDNA digestion activity as wild-type ExoX.
Figure S2.	Conservation of the DnaQ family active site.
Figure S3.	Variation in MeA-coordination in the active centres of the complex I and complex II.

Supplementary Table 1. Statistics of data collection and refinement

	Complex I Se-Met	Complex II	Complex III
Data collection			
Space group	$P2_12_12_1$	$P2_12_12_1$	$P2_12_12_1$
Cell dimensions			
a, b, c (Å)	33.5, 107.9, 117.1	37.3, 78.9,146.7	37.2, 79.0, 156.0
α, β, γ (°)	90.0, 90.0, 90.0	90.0, 90.0, 90.0	90.0, 90.0, 90.0
Wavelength (Å)	0.9789	1.000	1.000
Resolution (Å)	80-2.3	30-2.5	50-2.8
I/σ/	22.04(8.57)	29.09(9.87)	15.53(5.72)
R_{merge} (%)	12.2(28.4)	9.0(22.5)	12(46.6)
Completeness (%)	100(100)	99.8(97.8)	93.5(93.3)
Redundancy	11.9	7.0	6.9
Refinement			
Resolution(Å)	20-2.3	20-2.5	20-2.8
No. reflections	19692	15647	11945
R_{work}/R_{free} (%)	18.1/23.0	21.9/24.1	22.3/26.0
No. atoms			
Protein	2636	2636	2636
DNA	699	485	480
Water	149	23	30
B-factors (Å)	25.1	24.1	40.9
R.m.s. deviations			
Bond length (Å)	0.009	0.009	0.005
Bond angles (°)	0.938	0.842	0.688

Values in parentheses are for the highest shell

$R_{merge} = \sum|I - \langle I \rangle| / \sum I$, where I is the measured intensity for reflections with indices hkl.

$R_{work} = \sum||Fobs| - |Fcalc|| / \sum|Fobs.|$

R_{free} = R factor for a selected subset (10%) of the reflections that were not included in prior refinement calculations.

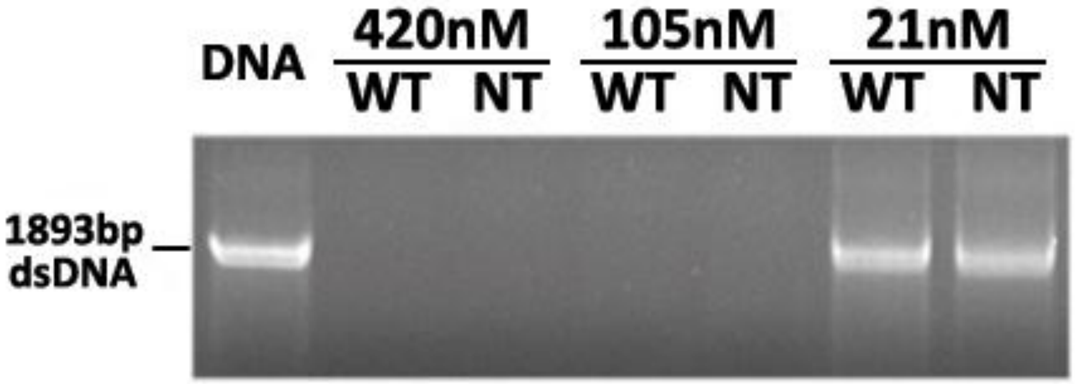

Supplementary Figure 1. Enzyme assay result showed NT (NT167, residues 1-167) has the same dsDNA digestion activity as wild type ExoX. The indicating concentration wild type and NT incubating with 32nM substrate double strand DNA under 37°C for 15min. The agarose gel was stained by EB.

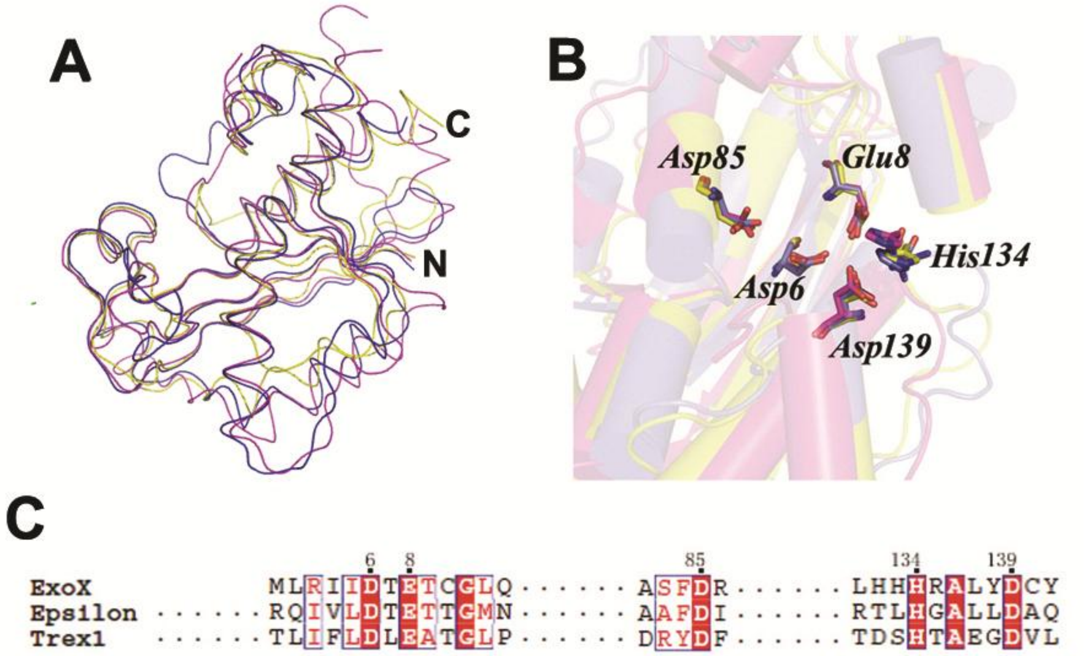

Supplementary Figure 2. Conservation of the DnaQ family active site. (A) Overall structure comparison of DnaQ members exonuclease core, ExoX (yellow), Epsilon (blue, PDB: 1J53), Trex1(magenta, PDB: 2OA8), suggested highly conservative folding. (B) Active site overlay of the structure of ExoX (yellow), Epsilon (blue) and Trex1 (magenta), showing the high similarity among the active centres. (C) Sequence alignment indicating the high degree of conservation between the key residues of ExoX, Epsilon (blue) and Trex1 (magenta).

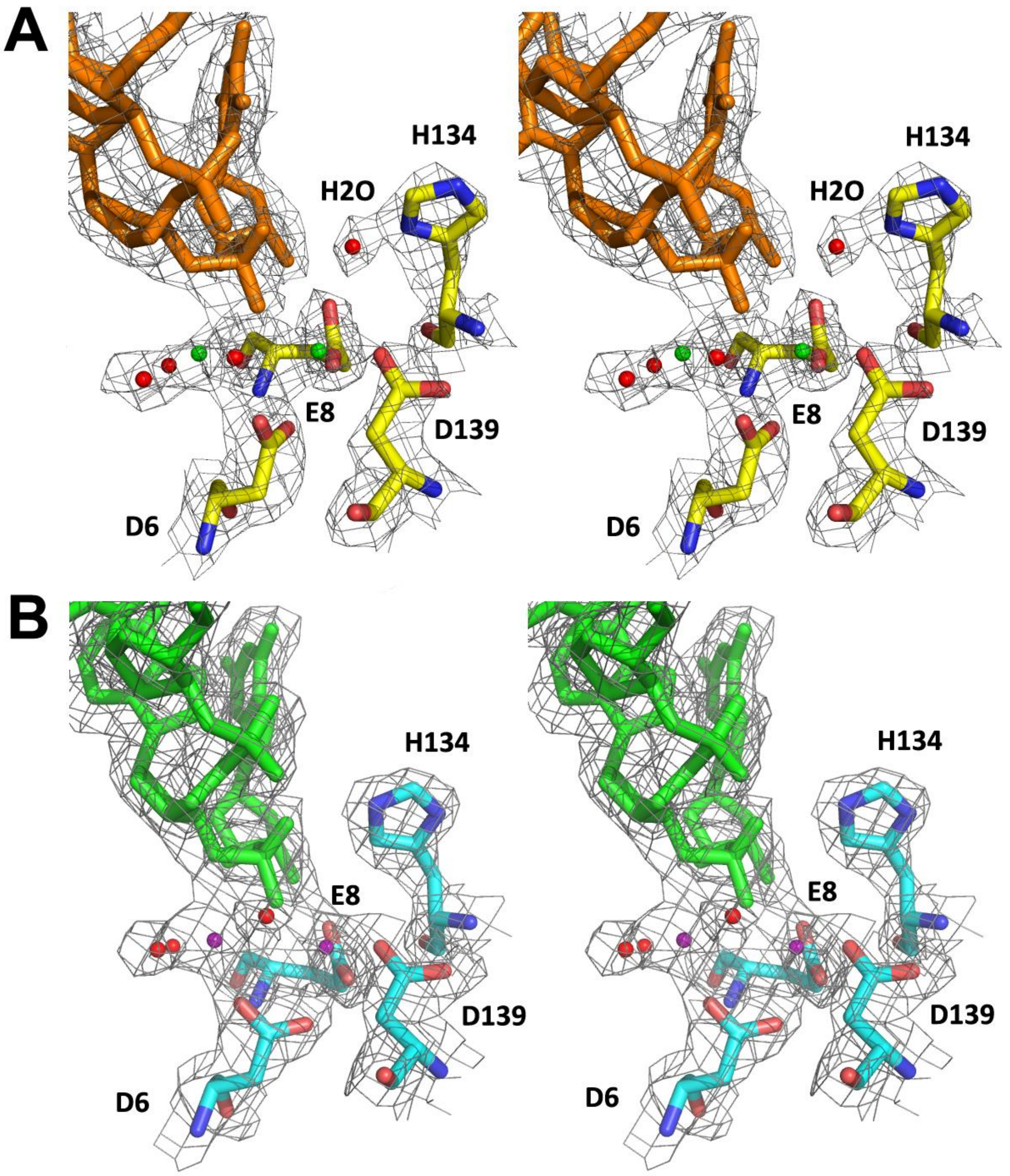

Supplementary Figure 3. Variation in MeA-coordination in the active centres of the complex I and complex II. The MeA is hexa-coordinated in the active centre of the complex I (A) and penta-coordinated in the complex II (B). A key solvent molecule is missing from the complex II. The density of the 2Fo-Fc omit map is contoured at 1.0 σ.
